# pH-sensitive probes for ischemic stroke: advancing early detection and accurate grading

**DOI:** 10.3389/fmolb.2026.1821099

**Published:** 2026-04-29

**Authors:** Chen Zou, Lijun Wang, Yue Wu, Pengfei Yang, Xianfu Meng, Jianmin Liu, Hongjian Zhang

**Affiliations:** 1 School of Health Science and Engineering, University of Shanghai for Science and Technology, Shanghai, China; 2 Oriental Pan-Vascular Devices Innovation College, University of Shanghai for Science and Technology, Shanghai, China; 3 Neurovascular Center, Changhai Hospital, Naval Medical University, Shanghai, China; 4 Department of Nuclear Medicine, Changhai Hospital, Shanghai Key Laboratory of Nautical Medicine and Translation of Drugs and Medical Devices, Navy Medical University, Shanghai, China; 5 Department of Materials Science and State Key Laboratory of Molecular Engineering of Polymers, Academy for Engineering and Technology, Fudan University, Shanghai, China

**Keywords:** early detection, ischemic stroke, metabolic imaging, pH-based grading, pH-responsive nanoprobes, tissue acidosis

## Abstract

Early detection and accurate assessment of ischemic stroke (IS) are essential for timely treatment and prognosis evaluation. Conventional bedside and imaging-based approaches, including the National Institutes of Health Stroke Scale (NIHSS), computed tomography (CT), and magnetic resonance imaging (MRI), remain central to acute stroke assessment, but they have important limitations in the hyperacute stage, when neurological deficits may be subtle and structural imaging changes may not yet be fully apparent. Among the metabolic alterations triggered by ischemia, tissue acidosis is particularly informative because pH declines rapidly after cerebral hypoperfusion and may reveal ischemic injury before conventional imaging abnormalities become obvious. In this review, we summarize recent advances in pH-based detection of IS, with emphasis on its potential for early diagnosis and physiologically informed stroke grading. We discuss current clinical and imaging assessment methods, the pathophysiological basis of ischemia-associated acidosis, emerging pH-detection technologies, and the major barriers to clinical translation. Finally, we outline a future perspective in which pH-related information is integrated with perfusion imaging, complementary biomarkers, and artificial intelligence to support a more comprehensive framework for ischemic stroke assessment.

## 1 Introduction

Ischemic stroke (IS), the most common subtype of stroke, remains a major cause of mortality, long-term disability, and healthcare burden worldwide. It accounts for approximately 87% of all stroke cases, underscoring the urgent need for timely diagnosis and intervention (Akinyemi et al., 2021; Zhang et al., 2023; Fan et al., 2023; Feigin et al., 2018). Because the efficacy of acute stroke therapies such as thrombolysis and mechanical thrombectomy is highly time-dependent, early and accurate assessment of stroke severity is essential for treatment selection and prognosis evaluation (Balami et al., 2013).

Conventional stroke assessment mainly relies on clinical scoring systems and structural imaging modalities. The National Institutes of Health Stroke Scale (NIHSS) is widely used for rapid neurological assessment (Kasner, 2006), while computed tomography (CT) and magnetic resonance imaging (MRI) play central roles in confirming stroke diagnosis, lesion localization, and treatment planning (Cui et al., 2022). However, these methods have important limitations in the hyperacute stage. Clinical scoring systems are influenced by symptom presentation and examiner experience, and may underestimate lesion severity in patients with subtle, evolving, or anatomically atypical deficits. Likewise, conventional imaging primarily captures structural and hemodynamic consequences of ischemia rather than the underlying metabolic disturbances that precede irreversible tissue injury (Leigh et al., 2018). As a result, current approaches are valuable for diagnosis and general severity assessment, but remain insufficient for capturing the early metabolic heterogeneity that determines tissue fate and therapeutic responsiveness.

Among the metabolic alterations triggered by ischemic stroke, tissue acidosis is one of the earliest and most informative events. Under physiological conditions, tissue pH serves as a key indicator of the local biochemical microenvironment (Chesler, 2003). Following interruption of cerebral blood flow, oxygen deprivation drives a shift from aerobic metabolism to anaerobic glycolysis, resulting in lactate accumulation and a rapid decline in local pH (Siesjö, 1984). Importantly, these pH alterations may precede many conventional imaging abnormalities, thereby providing an earlier window into ischemic injury. Furthermore, pH changes are not merely binary indicators of lesion presence; their magnitude, spatial distribution, and temporal evolution may reflect the severity of metabolic failure, the distinction between ischemic core and penumbra, and the potential for tissue recovery after reperfusion (Harston et al., 2015). Therefore, pH has the potential to support not only early detection but also a more physiologically grounded strategy for stroke grading.

In recent years, substantial progress has been made in pH-sensitive imaging strategies, including endogenous magnetic resonance-based approaches and exogenous probe-based platforms such as fluorescent, magnetic, and multimodal nanoprobes (Chen, 2021). These technologies have expanded the ability to visualize ischemia-associated acidosis with increasing sensitivity and spatial specificity. Nevertheless, three important gaps remain. First, the field still lacks a clear conceptual framework linking pH-derived signals to clinically meaningful stroke grading. Second, available pH detection technologies are often described individually rather than critically compared in terms of their suitability for early detection, quantitative grading, and translational deployment. Third, barriers to clinical implementation—including biosafety, pharmacokinetics, blood–brain barrier delivery, and outcome validation—have not been sufficiently integrated into current discussions. Addressing these issues is necessary if pH-sensitive imaging is to evolve from proof-of-concept visualization toward a practical tool for stroke stratification and precision management.

In this review, we first summarize conventional clinical and imaging assessment methods and the pathophysiological basis of tissue acidosis in IS. We then discuss the potential of pH alterations as a metabolic basis for stroke grading and critically evaluate current pH detection technologies with respect to their temporal sensitivity, spatial and quantitative performance, penetration depth, clinical feasibility, and translational readiness. Finally, we outline the major challenges and future directions for integrating pH-sensitive imaging with multimodal biomarkers and artificial intelligence to support a more dynamic and quantitative framework for acute stroke assessment.

## 2 Conventional clinical and imaging assessment of ischemic stroke

### 2.1 Clinical scoring systems

Clinical scoring systems remain the most widely used tools for the initial assessment of ischemic stroke severity because they are rapid, inexpensive, and readily applicable at the bedside. Among them, the NIHSS is the most commonly adopted instrument for quantifying neurological impairment in acute stroke. It evaluates multiple domains of neurological function, including level of consciousness, language, motor strength, sensory deficits, and coordination (Lyden et al., 1999; Shrestha et al., 2015). Individual item scores are assigned using ordinal scales and summed to a total score ranging from 0 to 42, with higher scores indicating more severe neurological dysfunction (Kwah and Diong, 2014). Owing to its simplicity and speed, the NIHSS plays an indispensable role in early triage, therapeutic decision-making, and outcome stratification in acute stroke care. Despite its clinical utility, the NIHSS has important limitations when used as a grading tool for ischemic brain injury. First, it is fundamentally a symptom-based scale and therefore reflects the clinical manifestation of stroke rather than the underlying biological or metabolic severity of tissue injury. Second, its performance depends on examiner experience and patient cooperation, which introduces inter-rater variability and may reduce reproducibility in emergency settings. Third, in the hyperacute stage, clinical deficits may still be subtle, evolving, or incompletely expressed. This is particularly relevant in posterior circulation stroke, cognitive dysfunction, or lesions affecting non-dominant networks, where the NIHSS may underestimate actual lesion burden and tissue risk (Heldner et al., 2013). To improve predictive performance, recent studies have explored combining NIHSS with circulating biomarkers. For example, serum occludin, a marker related to blood–brain barrier (BBB) disruption, has been reported to correlate with stroke severity and to improve the prediction of hemorrhagic transformation when used together with baseline NIHSS scores (Li et al., 2020; Yuan et al., 2021). However, although such strategies may enhance risk stratification, they do not resolve the central limitation of clinical scoring systems: they do not directly capture the metabolic state of ischemic brain tissue. Consequently, while clinical scales remain essential for rapid bedside evaluation, they are insufficient on their own to define the spatiotemporal heterogeneity of tissue injury during the hyperacute phase.

Although clinical scoring systems such as the NIHSS are indispensable for rapid bedside evaluation, they primarily reflect neurological impairment rather than the underlying metabolic or structural status of ischemic tissue. By contrast, imaging methods provide anatomical, vascular, and hemodynamic information that is critical for lesion localization and treatment planning. These categories are therefore complementary, but conceptually distinct, and this distinction is important when considering the role of pH-sensitive imaging as a metabolic bridge between clinical presentation and conventional structural imaging.

### 2.2 Imaging methods

Clinical imaging technologies are central to the diagnosis and assessment of acute ischemic stroke, with CT and MRI serving as the two major platforms for lesion detection, tissue characterization, and treatment planning. Compared with bedside clinical scoring systems, imaging provides objective visualization of lesion location and vascular status, thereby substantially improving diagnostic confidence in the acute setting.

CT is widely regarded as the first-line imaging modality in acute stroke because of its broad availability, high speed, and practical suitability for emergency workflows (Al Murdef et al., 2024). It is particularly valuable for excluding intracranial hemorrhage and identifying major structural abnormalities such as cerebral edema and large infarcts (Ho et al., 2012; Tomandl et al., 2003; Merino and Warach, 2010). In addition, CT perfusion (CTP) provides quantitative information on cerebral blood flow and can help delineate ischemic core–penumbra patterns, thereby supporting reperfusion decision-making (Vagal et al., 2019; Krishnan et al., 2017). However, the sensitivity of conventional CT to subtle ischemic changes in the hyperacute stage remains limited (Goldman, 2007; Gemmete, 2024). Although newer techniques such as spectral CT may improve lesion conspicuity and functional assessment (Mellander et al., 2023), CT-based approaches still primarily reflect structural and perfusion consequences of ischemia rather than the earliest metabolic disturbances.

MRI provides higher soft tissue contrast and greater sensitivity for early ischemic injury than CT, making it a powerful tool for acute stroke assessment and early lesion detection (Chizhik et al., 2014; Jin, 2018). The high soft tissue resolution and ability to provide hemodynamic data enhance MRI’s utility in stroke diagnosis (Baird and Warach, 1998; Merino and Warach, 2010). Diffusion-weighted imaging is considered the gold standard for early ischemia detection, as it can capture ischemic damage within minutes of onset (Dayani et al., 2017; Rordorf et al., 1998). Moreover, multi-sequence MRI techniques (e.g., T1, T2, FLAIR, SWI (Luo et al., 2018)) and perfusion-weighted imaging (PWI) can not only differentiate between hemorrhagic and ischemic stroke, but also quantify the extent of damage and evaluate areas of perfusion deficits (Rubin et al., 2022; Alaya et al., 2022). However, challenges such as high costs, prolonged scanning time, and the need for patient cooperation limit its use in emergency settings (Merino and Warach, 2010; Chaban et al., 2024). Additionally, motion artifacts in unstable patients can degrade image quality and delay diagnosis (You et al., 2021).

Taken together, CT and MRI are indispensable for stroke diagnosis, lesion localization, and treatment planning, but they remain imperfect tools for precise metabolic grading in the hyperacute stage. CT is efficient and widely accessible but relatively insensitive to early subtle ischemia, whereas MRI is more sensitive yet less practical in time-critical emergency scenarios. More importantly, even advanced structural and perfusion imaging does not directly quantify the biochemical deterioration that precedes irreversible tissue loss. This limitation is especially relevant when attempting to distinguish tissue that is already irreversibly damaged from tissue that is metabolically compromised but still potentially salvageable.

Therefore, there remains an unmet need for complementary approaches that move beyond symptom-based assessment and structural imaging to capture the metabolic severity and dynamic evolution of ischemic injury. In this context, pH-sensitive imaging is of particular interest because tissue acidosis is closely linked to early ischemic metabolism and may provide a more direct readout of tissue status during the hyperacute phase. By quantifying the degree and distribution of acidosis, pH-based approaches may help bridge the gap between bedside clinical presentation, conventional structural imaging, and a more physiologically grounded assessment of ischemic stroke severity.

## 3 Principles and physiological mechanisms of pH detection

### 3.1 pH changes in acute ischemic stroke

**FIGURE 1:**
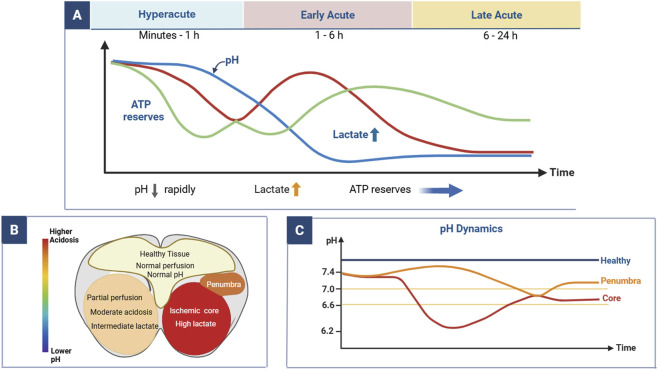
Spatiotemporal evolution of tissue acidosis in ischemic stroke. **(A)** Temporal changes in pH, lactate levels, and ATP reserves during hyperacute, early acute, and late acute phases. **(B)** Spatial distribution of pH and metabolic alterations across healthy tissue, penumbra, and ischemic core. **(C)** Dynamic pH changes over time in healthy tissue, penumbra, and ischemic core.

Upon the occurrence of ischemic stroke (Figure 1), a complex set of pathophysiological processes is initiated and ultimately leads to brain injury (Dirnagl et al., 1999; Song et al., 2017). One of the earliest and most prominent metabolic consequences is the development of acidosis (Allen and Bayraktutan, 2009; Raichle, 1983). This acidosis primarily arises from a shift in cellular metabolism induced by oxygen deprivation (Nathaniel et al., 2015; Hu and Song, 2017). Under physiological conditions, brain cells rely predominantly on aerobic metabolism to generate adenosine triphosphate (ATP) through oxidative phosphorylation (Whittingham, 1990; Zhou et al., 2021; Zauner et al., 2002). However, when ischemia occurs and oxygen availability declines, cells are forced to switch to anaerobic glycolysis in order to maintain energy production (Liang et al., 2021). In this state, glucose is metabolized into lactate rather than carbon dioxide and water, resulting in the accumulation of lactic acid within ischemic tissue (Mahan, 2021; Wass and Lanier, 1996). This metabolic shift disrupts normal acid–base homeostasis and drives a rapid decline in local pH (TóTHO. et al., 2020). The physiological pH of brain tissue normally ranges from 7.04 to 7.20 (Rehncrona, 1985), but this balance is markedly disturbed during ischemia as lactate accumulates and proton clearance becomes impaired (Raichle, 1983; Sarkar et al., 2019).

The degree of acidification is not spatially uniform, but varies across different ischemic regions. In the ischemic core, where blood flow is severely reduced or completely interrupted, oxygen deprivation is maximal and anaerobic metabolism becomes dominant (Tóth, 2021; Cheung et al., 2021). As a result, pH declines most profoundly in this region, reflecting severe metabolic collapse and rapid progression toward irreversible injury (Hu and Song, 2017; Dienel and Hertz, 2005). In contrast, the penumbra surrounding the ischemic core retains partial perfusion, which allows a limited degree of aerobic metabolism to persist (Davis and Donnan, 2021; Ermine et al., 2021). Consequently, the pH in the penumbra decreases less markedly than in the core, typically remaining around 6.70 (Choi, 1990). This spatial pH heterogeneity is biologically important because it mirrors regional differences in tissue viability and metabolic reserve.

The severity of tissue acidosis also evolves dynamically over time. During the hyperacute stage of ischemic stroke, which occurs within minutes to hours after onset, pH declines rapidly in the affected tissue (Heo et al., 2023; Dijkhuizen et al., 1998). In the ischemic core, pH may fall to approximately 6.0 due to the swift accumulation of lactate and failure of energy metabolism (Tietze et al., 2014; Heo et al., 2023; Dijkhuizen et al., 1998; Larkin et al., 2021). This severe acidification contributes to excitotoxicity, calcium overload, oxidative stress, and mitochondrial dysfunction, thereby amplifying neuronal injury (Kristián and Siesjö, 1998; Taoufik and Probert, 2008; Radak et al., 2014; Ludhiadch et al., 2022; Bretón and Rodríguez, 2012; Sims and Anderson, 2002). During the acute stage, typically spanning hours to days after ischemia, partial compensation may occur through collateral perfusion, lactate clearance, and limited metabolic recovery (Brouns et al., 2011; Gauthier and Szerlip, 2002). As a consequence, pH in the ischemic core may increase modestly, although it generally remains below normal values. A similar but less severe pattern is observed in the penumbra, where pH recovery may occur if perfusion is restored in time (Wang et al., 2019).

In addition to reflecting metabolic failure, acidosis actively participates in ischemic injury progression. Acidic conditions exacerbate excitotoxicity, disrupt intracellular calcium homeostasis, impair protein synthesis, and weaken blood–brain barrier integrity, thereby promoting edema formation, inflammatory infiltration, and hemorrhagic transformation (Gerriets et al., 2009; Sarkar et al., 2019; Hu and Song, 2017). This acidosis further disrupts protein synthesis, impairing the capacity for cellular self-repair and the maintenance of normal functions (Allen and Bayraktutan, 2009, Kristián and Siesjö, 1998). Therefore, pH changes are not merely by-products of ischemia, but integral components of the evolving ischemic microenvironment.

Overall, acute ischemic stroke is characterized by a distinct spatiotemporal pattern of tissue acidification, with more severe acidosis in the ischemic core, milder but clinically relevant acidosis in the penumbra, and dynamic temporal evolution from the hyperacute to the acute phase. This pattern provides an important physiological basis for using pH as an indicator of ischemic tissue status.

### 3.2 Early diagnostic and therapeutic significance of pH changes

Following ischemic stroke, the rapid drop in pH reflects the metabolic shift and severity of ischemic injury, thereby creating a critical window for early diagnosis and intervention. During the hyperacute stage, the ischemic core undergoes profound acidification, whereas the penumbra exhibits a relatively milder reduction in pH (Farkas and Rose, 2024). These regional differences are highly relevant because they may help distinguish tissue that is already severely damaged from tissue that remains metabolically compromised but potentially salvageable (Demeestere et al., 2020). Accordingly, pH alterations provide more than a generic signal of ischemia; they may also offer insight into tissue viability and the urgency of therapeutic intervention.

pH monitoring offers a significant advantage in this context because it can detect metabolic abnormalities before overt anatomical changes become apparent on conventional imaging. Advanced methods such as magnetic resonance spectroscopy and pH-sensitive probes enable visualization of pH dynamics *in vivo*, thereby providing a sensitive marker for ischemia during its earliest stages. This early metabolic sensitivity is particularly important in the hyperacute phase, when treatment decisions must often be made before structural injury has fully evolved. In this setting, pH-based information may complement conventional imaging by identifying tissue at risk, refining estimation of the ischemic penumbra, and improving the physiological interpretation of lesion severity.

Beyond early diagnosis, pH monitoring may also be useful for evaluating treatment response and tracking the evolution of ischemic injury. During the acute phase, when reperfusion or collateral circulation partially restores tissue oxygenation, pH may recover to some extent as lactate is cleared and aerobic metabolism is partially re-established (Huber and Heiss, 1996). A rise in pH after reperfusion may indicate metabolic recovery and tissue rescue, whereas persistently low pH may suggest ongoing ischemia, failed reperfusion, or progression toward irreversible infarction. Thus, dynamic pH assessment has the potential to provide longitudinal information that is not fully captured by static structural imaging alone.

Importantly, the significance of pH changes extends beyond simple lesion detection. Because acidosis varies in depth, extent, regional distribution, and reversibility, pH-related information may help stratify ischemic severity more precisely than binary classifications based solely on lesion presence or absence. For example, the intensity of acidification may reflect the severity of metabolic collapse, the spatial distribution of low-pH regions may help differentiate ischemic core from penumbra, and the trajectory of pH recovery may provide insight into treatment responsiveness and prognosis (Foo et al., 2021). These features suggest that pH has the potential to serve not only as an early diagnostic marker, but also as a biologically meaningful basis for stroke grading.

Accordingly, the clinical value of pH monitoring lies not only in detecting ischemic tissue, but also in quantifying its metabolic state and dynamic evolution. This provides the conceptual foundation for a pH-based grading framework, in which measurable aspects of acidosis can be linked to tissue fate, therapeutic response, and clinical outcome.

### 3.3 pH-based grading framework for ischemic stroke

Although tissue acidosis is widely recognized as an early metabolic feature of ischemic stroke, its significance may extend beyond lesion detection alone. Conceptually, pH alterations may also be considered in relation to stroke grading, as they reflect metabolic disturbances that are not fully captured by clinical scoring systems or conventional structural imaging (Tietze et al., 2014). In this context, a pH-oriented grading framework would not replace existing methods, but could provide an additional metabolic dimension for assessing ischemic tissue status.

Such a framework should not rely on a single absolute pH value. Instead, the grading relevance of pH may be understood in terms of several related features, including the severity, spatial extent, regional distribution, and temporal evolution of tissue acidosis. Several pH-related parameters may therefore be of interest. Minimum lesion pH may reflect the degree of metabolic failure in the most severely affected tissue (Harston et al., 2015). Acidosis volume may indicate the overall burden of acidified tissue (Heo et al., 2017). The pH gradient between ischemic core and penumbra may help describe metabolic heterogeneity within the lesion (Tietze et al., 2014). The temporal profile of pH change, particularly pH recovery after reperfusion, may also provide information on whether metabolic dysfunction is persistent or partially reversible (Heo et al., 2023).

Importantly, these parameters should still be regarded as candidate variables rather than established grading criteria. Their value depends on whether they can be reproducibly measured and meaningfully associated with infarct progression, reperfusion response, and functional outcome (Huang et al., 2015). From this perspective, the key issue is not only whether a given technique can detect acidosis, but also what aspect of acidosis it can characterize, and with what degree of spatial, temporal, and quantitative reliability.

Accordingly, the following sections discuss pH-sensitive technologies not only in terms of ischemia detection, but also in relation to the type of pH-related information they may provide for a more refined assessment of ischemic stroke severity.

## 4 pH detection methods in ischemic stroke and their application in stroke grading

The significance of pH detection in ischemic stroke lies not only in identifying lesion-associated acidosis, but also in characterizing the metabolic severity and spatial heterogeneity of ischemic tissue. In contrast to conventional grading methods, which mainly rely on neurological deficits or structural abnormalities, pH-sensitive approaches may provide information that is more directly related to tissue metabolism during the hyperacute and acute phases. Accordingly, different pH detection technologies should be evaluated not only by whether they can detect acidosis, but also by what type of pH-related information they can provide and how relevant that information may be for early detection and stroke grading.

In this section, these technologies are compared with respect to their temporal responsiveness for early detection, spatial and quantitative relevance for grading, penetration depth, technical and clinical feasibility, and current translational readiness.

### 4.1 Traditional pH detection methods (non-probe techniques)

#### 4.1.1 *Ex vivo* techniques

Monitoring tissue pH is fundamental to ischemic stroke research, as acidosis directly reflects metabolic failure following cerebral hypoperfusion. A variety of *ex vivo* strategies have therefore been developed to characterize pH alterations in ischemic brain tissue.

Simple methods, including litmus paper and multirange indicator strips, provide rapid and inexpensive estimates of acidity but are restricted to qualitative or semi-quantitative assessment (Walpole, 1913; Gamble and Evers, 1922; Gomez et al., 2024; Khan et al., 2017; Power et al., 2017; Steinegger et al., 2020). For higher precision, glass-electrode pH meters are widely used to quantify hydrogen ion activity in tissue homogenates and *in vitro* samples. Although these instruments offer excellent accuracy and temporal resolution, their measurements are highly sensitive to temperature, electrode stability, and gas exposure, and they remain fundamentally limited to *ex vivo* analysis (Dankwa, 2020; Ghoneim et al., 2019; Cheng and Zhu, 2005; Cheng et al., 1998).

To obtain spatial information, histochemical staining techniques such as neutral red have been introduced to visualize regional tissue acidosis in brain sections (Kogure et al., 1980). Fluorescence-based histochemical methods further improve spatial resolution and permit more quantitative reconstruction of pH distributions across ischemic and non-ischemic tissue regions (Csiba et al., 1983). However, despite these methodological advances, both colorimetric and fluorescence-based *ex vivo* methods remain limited by their dependence on excised or post-mortem tissue, which restricts their value for real-time clinical assessment.

Overall, *ex vivo* techniques are valuable for mechanistic studies and for validating pH changes at the tissue level, but their contribution to stroke grading is mainly indirect. They can help define the existence, extent, and microscopic distribution of acidosis, yet they cannot provide continuous *in vivo* monitoring or support time-sensitive decision-making in acute clinical settings. Therefore, while these methods are important for establishing the biological basis of ischemic acidosis, they have limited translational value as stand-alone tools for clinical stroke grading.

#### 4.1.2 *In vivo* endogenous imaging techniques

Traditional *in vivo* imaging methods, particularly magnetic resonance-based techniques, have played a central role in assessing pH-related changes during ischemic stroke. Unlike *ex vivo* assays, these approaches allow non-invasive interrogation of tissue metabolism and therefore offer greater potential for dynamic stroke assessment. Their relevance to stroke grading depends not only on whether they can detect acidosis, but also on how well they capture lesion-level heterogeneity, temporal evolution, and clinically actionable metabolic information.

Among them, magnetic resonance spectroscopy (MRS) provides metabolic information by detecting nucleus-specific spectral signals (Wu et al., 2023). Phosphorus-31 magnetic resonance spectroscopy (^31^P-MRS) is particularly notable because it enables relatively direct estimation of intracellular pH based on the chemical shift of inorganic phosphate relative to phosphocreatine (Seo et al., 1983). In a seminal study, Germano and colleagues applied ^31^P-MRS in a rat model of focal ischemic stroke and showed that intracellular pH rapidly decreased after arterial occlusion, supporting the view that acidification is an early event in ischemic injury (Germano et al., 1988). This finding highlights the strength of ^31^P-MRS as a method for probing early metabolic changes before overt structural damage develops. However, its relatively low spatial resolution and limited practicality constrain its broader application in precise lesion stratification.

Proton MRS (^1^H-MRS) has also been used to assess stroke-associated metabolic changes, particularly through metabolites such as lactate, N-acetylaspartate, creatine, and choline. Compared with ^31^P-MRS, ^1^H-MRS offers higher sensitivity and improved spatial resolution, and can indirectly reflect ischemia-associated acidification through lactate accumulation (Vial et al., 2004). Nevertheless, because pH is inferred rather than directly mapped, its value for quantitative pH-based grading remains limited. Even so, ^1^H-MRS remains informative for identifying lactate-associated metabolic stress and therefore retains value as a complementary method for ischemia-related biochemical assessment.

Amide proton transfer (APT) MRI, a chemical exchange saturation transfer (CEST)-based technique, has emerged as a particularly promising endogenous pH-sensitive imaging approach. APT MRI exploits the pH dependence of proton exchange between endogenous amide groups and water, allowing pH-weighted imaging with substantially higher spatial resolution than MRS (Ray, 2017). Recent studies have shown that ratiometric and multipool APT approaches can improve pH specificity and better differentiate ischemic tissue compartments in acute stroke models (Wu and Sun, 2023; Jin et al., 2024). Compared with MRS, APT MRI is better suited to lesion-wide assessment and to depicting spatial heterogeneity between ischemic core, infarct growth regions, and surrounding oligemic tissue. This feature makes APT MRI especially relevant when considering the possible use of pH information for stroke grading.

Taken together, non-probe *in vivo* techniques provide the most clinically realistic route for endogenous pH assessment, but each approach involves distinct trade-offs. ^31^P-MRS offers relatively direct metabolic information and is valuable for probing the severity of acidification in the most affected tissue, but its spatial resolution remains insufficient for lesion-level grading. ^1^H-MRS is more sensitive and experimentally accessible, yet its pH information remains indirect and is therefore better suited to reflecting lactate-associated metabolic stress than to supporting quantitative pH-based grading. By comparison, APT MRI provides improved spatial mapping and appears more compatible with lesion-level grading, particularly where the spatial distribution of acidosis and heterogeneity across ischemic regions are of interest, although it still faces challenges related to acquisition time, standardization, and susceptibility to confounding effects (Zheng and Wang, 2021; Geng et al., 2010; Santos-Díaz and Noseworthy, 2020). Therefore, among current non-probe methods, APT MRI may currently be the most relevant for refined pH-based grading of ischemic stroke, although further technical validation remains necessary before it can be regarded as a robust grading tool.

### 4.2 Emerging pH detection technologies (probe-based techniques)

#### 4.2.1 *Ex vivo* probe-based techniques

Accurate monitoring of pH dynamics in brain tissue is essential for elucidating ischemic stroke pathophysiology and evaluating therapeutic efficacy. Conventional *ex vivo* methods, including glass-electrode pH meters and indicator papers, provide only averaged measurements and lack the spatial resolution, sensitivity, and temporal responsiveness required to capture rapid microscale pH fluctuations during ischemic progression. Unlike these bulk assays, probe-based *ex vivo* techniques are primarily designed to improve microscale spatial sensitivity and localized pH responsiveness. Consequently, nanotechnology-enabled sensing strategies, particularly surface-enhanced Raman spectroscopy (SERS) probes, have attracted growing interest as high-precision alternatives.

Laurence and colleagues introduced a silver nanoparticle (AgNP)-based SERS pH sensor using 4-mercaptobenzoic acid (4-MBA) as a molecular reporter, enabling quantitative pH estimation through ratiometric analysis of pH-dependent Raman spectral shifts (Laurence et al., 2004). This nanoscale strategy demonstrated the feasibility of localized pH sensing beyond bulk tissue measurements. However, its translational potential was limited by nanoparticle aggregation-induced signal instability, shallow tissue penetration under visible-light excitation, and temporal fluctuations caused by Brownian motion.

Subsequent efforts sought to improve signal stability and optical performance. For example, structurally optimized plasmonic nanosystems were developed to enhance electromagnetic field confinement and enable near-infrared excitation, thereby reducing background interference and modestly improving penetration depth 00000000000did not fundamentally resolve the major limitations of *ex vivo* nanosensors, namely restricted tissue accessibility and limited applicability to deep-brain, real-time monitoring.

Beyond SERS, nanoplasmonic resonance energy transfer (PRET) spectroscopy offers another high-precision *in vitro* strategy for pH-sensitive imaging (Liu, 2009). By detecting resonance-induced dips in nanoparticle scattering spectra, PRET can achieve submicrometre spatial resolution and high pH sensitivity. Nevertheless, its dependence on highly controlled optical conditions currently confines it to experimental settings.

Overall, probe-based *ex vivo* nanosensors substantially improve the spatial precision of pH measurement compared with conventional bulk assays, and they are useful for mechanistic interrogation of microscale acidosis. However, their relevance to stroke grading remains limited because they do not provide clinically deployable, lesion-level, or longitudinal assessment. Thus, although these techniques offer excellent microscale spatial sensitivity, their limited penetration depth, *ex vivo* dependence, and lack of clinical feasibility make them unsuitable for real-time stroke grading in practice.

#### 4.2.2 *In vivo* probe-based techniques

**FIGURE 2:**
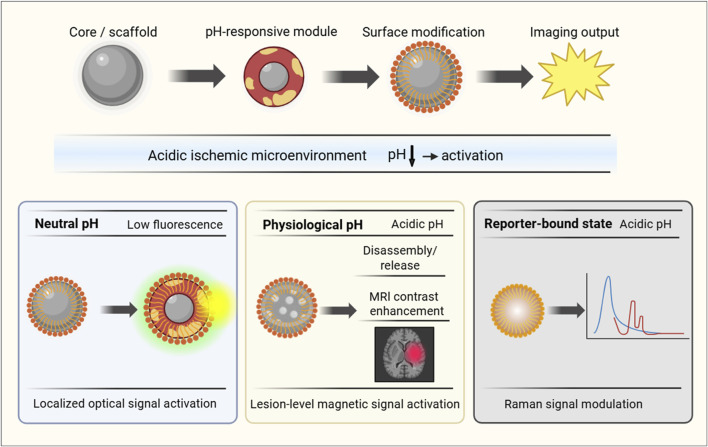
General design and activation mechanisms of pH-responsive nanoprobes. The schematic summarizes the common architectural elements of pH-responsive nanoprobes and three representative activation modes under acidic conditions relevant to ischemic stroke: fluorescence turn-on, MRI contrast activation or contrast-agent release, and SERS spectral shift.

The *in vivo* detection of stroke-associated acidosis has attracted particular attention because it offers the possibility of directly visualizing metabolic abnormalities within living brain tissue. Unlike endogenous MRI-based techniques, probe-based strategies are designed to enhance pH sensitivity, amplify contrast changes, or integrate multiple imaging functions. Their main advantage lies in their engineering flexibility: probe composition, targeting capability, signal mechanism, and imaging modality can all be tuned to match specific biological and diagnostic needs. At the same time, this flexibility introduces substantial challenges related to biosafety, delivery, and clinical translation. Accordingly, their value in stroke management depends not only on pH sensitivity but also on temporal responsiveness, lesion-level quantification, penetration depth, and translational feasibility. A further issue that must be considered for all systemically administered probe-based strategies is how they reach ischemic brain tissue despite vascular occlusion and the partial preservation of the blood–brain barrier. In practice, probe accumulation is likely to depend on a combination of factors, including residual regional blood flow, stroke-induced BBB disruption, circulation time, and active targeting mechanisms. This also implies that delivery may be spatially heterogeneous, with greater probe access in partially perfused or BBB-compromised regions than in severely ischemic core tissue. Accordingly, signal generation *in vivo* reflects not only pH responsiveness itself, but also the efficiency and timing of probe delivery to the lesion. The general design logic and representative activation mechanisms of pH-responsive nanoprobes are summarized in Figure 2. Representative examples of pH-responsive nanoprobes and their material and pharmacokinetic characteristics are summarized in Table 1.

**TABLE 1:** Material and pharmacokinetic properties of representative pH-responsive nanoprobes.

Probe/Reference	Core material	Surface coating/Formulation	Reported biocompatibility evidence
CdSe@ZnS/ZnS QDs-loaded micelles (Yang et al., 2017)	CdSe@ZnS/ZnS quantum dots	Polymeric micelles	Heavy-metal safety concern remains
aza-BODIPY liposomal probe (Yao et al., 2023)	Organic aza-BODIPY fluorophore	Liposomal formulation	BBB penetration reported; potentially improved biosafety
VCAM-1-targeted NIR-IIb nanoprobe (Yang et al., 2021)	Activatable NIR-IIb nanoprobe	VCAM-1-targeted formulation	High-contrast lesion visualization reported
PEG–PAEA/Fe_3_O_4_micelles (Gao et al., 2011)	Fe_3_O_4_nanoparticles	PEG–PAEA micelles	Acid-responsive activation demonstrated
Biodegradable Fe_3_O_4_micelles (Yang et al., 2016)	Fe_3_O_4_nanoparticles	Biodegradable polymeric micelles	Improved biosafety profile reported
RAPA/Gd^3+^@NPs (Cheng et al., 2021)	Gd^3+^-containing nanoparticles	Theranostic nanoparticle formulation	Multimodal imaging demonstrated
MnCO_3_@BSA–ICG nanoparticles (Song et al., 2022)	MnCO_3_+ ICG	BSA-based nanoparticle system	Improved biocompatibility reported

##### 4.2.2.1 pH-responsive fluorescent probes

Fluorescence imaging has emerged as a powerful modality for stroke research because of its high sensitivity, real-time capability, and relatively high spatial resolution. Unlike CT or MRI, fluorescence imaging enables dynamic visualization of molecular and metabolic processes. However, conventional fluorescence imaging is limited by shallow tissue penetration, photobleaching, and non-specific background signal. Because ischemic stroke is characterized by rapid local acidification, pH-responsive fluorescent probes can directly visualize this metabolic abnormality *in vivo*.

Yang and colleagues reported a pH-sensitive probe based on CdSe@ZnS/ZnS quantum dots encapsulated in polymeric micelles, which exhibited pH-dependent fluorescence modulation over a physiologically relevant range and preferential accumulation in ischemic brain tissue, enabling visualization of metabolic acidosis *in vivo* (Yang et al., 2017). This study illustrates the major strength of inorganic fluorescent nanoprobes, namely, strong signal responsiveness and high sensitivity. However, the use of heavy-metal-containing materials raises important concerns regarding long-term biocompatibility and translational safety.

To address this issue, organic fluorophore-based systems have emerged as a more biocompatible alternative. Yao and co-workers developed a pH-responsive NIR-I liposomal probe based on aza-BODIPY derivatives that crossed the disrupted blood–brain barrier and produced acidity-activated fluorescence correlated with neurological deficit severity (Yao et al., 2023). Compared with inorganic quantum-dot systems, organic probes may offer improved biosafety and translational compatibility, but they still face limitations in tissue penetration and quantitative robustness.

To further improve penetration depth and signal-to-noise ratio, NIR-II fluorescence imaging extends optical detection into deeper tissue while improving signal-to-noise characteristics. An activatable VCAM-1-targeted NIR-IIb nanoprobe enabled high-contrast visualization of inflamed cerebral vasculature and early ischemic lesions, with signal intensity reflecting the extent of oxidative stress and vascular injury (Yang et al., 2021). Although this platform was not purely a pH reporter, it illustrates the broader value of activatable deep-tissue optical probes for improving lesion conspicuity in early stroke. From a grading perspective, fluorescent probes are attractive because they can sensitively detect localized acidosis and may provide dynamic information, but their utility is still constrained by penetration depth, signal attenuation, and the difficulty of achieving robust lesion-wide quantification in deep brain tissue. Their *in vivo* performance therefore depends not only on pH-triggered signal activation, but also on whether sufficient probe delivery can be achieved in partially perfused or BBB-disrupted tissue compartments.

Taken together, fluorescent probes provide excellent temporal responsiveness and high sensitivity for early metabolic detection, making them attractive for dynamic monitoring of localized acidosis. However, their limited penetration depth, reduced lesion-wide quantitative robustness in deep brain tissue, and unresolved biosafety and delivery concerns currently restrict their utility for comprehensive clinical stroke grading. Their *in vivo* performance therefore depends not only on pH-triggered signal activation, but also on whether sufficient probe delivery can be achieved in partially perfused or BBB-disrupted tissue compartments. This means that strong probe-level fluorescence responsiveness does not automatically ensure robust lesion-level detection efficiency, particularly in deep brain tissue where penetration and signal attenuation remain limiting factors. In practice, their greatest value may lie in identifying early focal acidification and in tracking dynamic pH changes over time, whereas their contribution to whole-lesion assessment remains more limited. In addition, NIR-II systems may offer advantages over NIR-I probes in penetration depth, whereas organic formulations may be more favorable than inorganic quantum-dot systems in terms of translational safety.

##### 4.2.2.2 pH-responsive MRI probes

Traditional MRI techniques are highly effective for anatomical imaging but cannot detect real-time metabolic changes associated with ischemic stroke progression (Vasireddi et al., 2024). By incorporating pH-responsive contrast mechanisms, MRI can potentially be extended from structural assessment to metabolism-sensitive imaging.

A representative example is a PEG–poly (β-amino ester)/(amido amine) (PEG–PAEA) micellar system encapsulating Fe_3_O_4_ nanoparticles, which remains stable under physiological conditions but undergoes rapid disassembly in acidic environments (Gao et al., 2011). This pH-triggered destabilization accelerates Fe_3_O_4_ release at pH values ≤6.8, corresponding to those observed in ischemic tissue, and enhances local transverse relaxivity, thereby amplifying T_2_-weighted contrast in acidified regions. The principal advantage of this strategy is that it links MRI signal activation directly to the acidic microenvironment, thereby improving lesion selectivity compared with non-responsive contrast enhancement. However, rapid release kinetics may shorten the imaging window, and long-term iron oxide retention remains a concern.

To address these limitations, an alternative strategy uses biodegradable polymeric micelles decorated with Fe_3_O_4_ nanoparticles to favor gradual rather than abrupt release (Yang et al., 2016). In this design, gradual nanoparticle release prolonged lesion visibility and allowed more continuous monitoring of infarct evolution. Compared with abrupt release systems, such biodegradable platforms may be better aligned with the temporal dynamics of ischemic injury and may offer improved biosafety profiles. This makes pH-responsive MRI probes conceptually attractive for stroke grading, because they combine lesion-level spatial information with a pH-triggered functional readout. For these systems, lesion selectivity depends not only on pH-triggered contrast activation, but also on whether the probe can reach acidified tissue in sufficient amounts within the relevant ischemic time window. In this context, translational value depends not only on pH selectivity but also on whether contrast activation can be achieved without unacceptable retention, toxicity, or loss of imaging stability.

Nevertheless, substantial translational challenges remain, including nanoparticle retention, uncertain long-term safety, delivery efficiency, and the need to balance pH responsiveness with imaging stability. Compared with endogenous approaches such as APT-MRI, pH-responsive MRI probes are currently less mature for clinical implementation. However, they remain conceptually important because they directly couple signal activation to the acidic microenvironment and may therefore offer stronger pH specificity in lesion-level assessment. This feature makes them particularly attractive where the spatial distribution and overall burden of acidosis are of interest, although their translational readiness remains lower than that of endogenous MRI-based approaches.

##### 4.2.2.3 pH-responsive multimodal probes

Although pH-responsive MRI probes extend conventional imaging beyond anatomical contrast, they provide limited real-time functional information during ischemic stroke progression. To overcome this constraint, multimodal platforms combine MRI with near-infrared fluorescence (NIRF) or photoacoustic imaging (PAI), thereby enabling complementary anatomical and functional readouts.

Cheng and colleagues reported a pH-sensitive theranostic nanoparticle system (RAPA/Gd^3+^@NPs) that integrates acid-triggered drug release with MRI and NIRF imaging capability (Cheng et al., 2021). Under mildly acidic conditions characteristic of ischemic tissue, the particles released rapamycin while simultaneously enhancing T_1_-weighted MRI contrast and activating NIRF signals. This design enabled visualization of ischemic regions before overt structural damage and allowed signal intensity to serve as an indirect indicator of metabolic severity. The value of such multimodal systems lies in their ability to couple lesion localization with metabolic sensing and, in some cases, therapy delivery. However, concerns related to gadolinium-associated toxicity, system complexity, and the limited penetration depth of NIRF remain important barriers.

Song and co-workers introduced an alternative MRI–PAI platform based on MnCO_3_@BSA–ICG nanoparticles (Song et al., 2022). Compared with gadolinium-based systems, manganese-containing platforms may offer improved biocompatibility and reduced long-term deposition risk. In addition to enhancing MRI contrast, these nanoparticles enabled photoacoustic monitoring of cerebral oxygen saturation, thereby providing complementary information on ischemia-associated metabolic compromise. Compared with NIRF, photoacoustic imaging offers deeper tissue penetration and may therefore be better suited for interrogating deeper lesions. However, the addition of multiple modalities also increases technical complexity, manufacturing demands, and barriers to standardization.

Overall, multimodal pH-responsive probes are attractive because they can integrate anatomical, metabolic, and functional information within a single platform, making them valuable for preclinical studies and for exploring more comprehensive models of ischemic tissue assessment. From a grading perspective, their main advantage lies in combining lesion localization, metabolic sensing, and complementary physiological readouts, which may be particularly useful when pH is interpreted alongside oxygenation, perfusion, or other indicators of tissue status. However, this gain in information richness is offset by greater technical complexity, more difficult standardization, and weaker current translational readiness. Accordingly, although multimodal probes may provide richer biological information than single-modality systems, their role in clinical stroke grading remains largely exploratory.

#### 4.2.3 Comparative perspective on probe-based pH detection technologies

**FIGURE 3:**
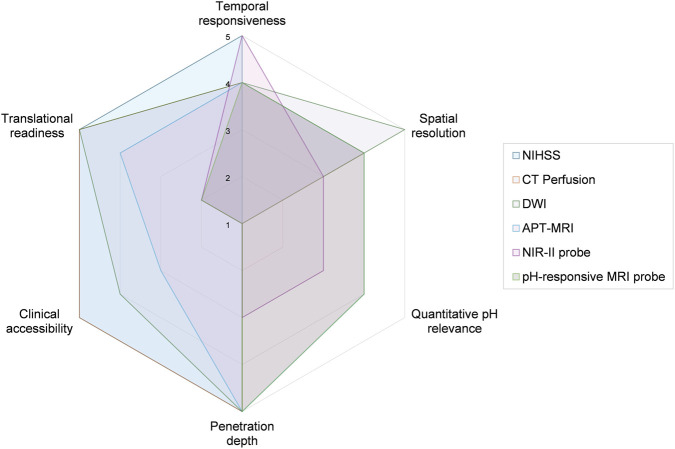
Performance radar chart of key pH-detection technologies. The chart provides a semi-quantitative comparison of selected representative approaches, including NIHSS, CT perfusion, DWI, APT-MRI, NIR-II probes, and pH-responsive MRI probes, across dimensions relevant to ischemic stroke assessment: temporal responsiveness, spatial resolution, quantitative pH relevance, penetration depth, clinical accessibility, and translational readiness. NIHSS, CT perfusion, and DWI are included as clinical reference comparators rather than pH-specific techniques.

From a comparative perspective, probe-based pH detection strategies exhibit distinct strengths and limitations in stroke management. Fluorescent probes offer the strongest temporal responsiveness and signal sensitivity for early metabolic detection, but remain constrained by penetration depth and limited lesion-wide quantification. pH-responsive MRI probes are more compatible with lesion-level spatial assessment and may be more suitable for metabolism-oriented grading, yet they face substantial challenges in biosafety, delivery, and imaging stability. Multimodal probes provide the richest combination of anatomical, metabolic, and functional information, but their increased complexity currently limits translational readiness. Importantly, improved signal responsiveness at the probe level does not necessarily translate into superior detection efficiency *in vivo*, because clinical utility depends on adequate tissue delivery, sufficient penetration, low background interference, and the ability to generate reliable lesion-wide information. Taken together, probe-based technologies are particularly valuable for expanding sensitivity, activatable contrast, and functional integration, although their current clinical feasibility generally remains lower than that of endogenous MRI-based approaches. Accordingly, future progress in pH-based stroke grading will likely depend not on a single dominant platform, but on balancing metabolic sensitivity, spatial precision, penetration depth, clinical practicality, and translational safety. To facilitate representative cross-platform comparison, Figure 3 summarizes the relative strengths and limitations of selected stroke assessment and pH-sensitive technologies in ischemic stroke. A comparative overview of different pH-detection technologies and their clinical translation potential is provided in Table 2.

**TABLE 2:** Comparative analysis of pH-detection technologies for stroke: clinical translation potential.

Technology	Key strength for stroke	Primary clinical limitation	TRL (1–9)	Major translational hurdle
*Ex vivo* pH assays	Accurate tissue-level validation	No real-time *in vivo* assessment	2	Inherently *ex vivo*
MRS-based methods	Metabolic interrogation	Low spatial specificity	4–5	Limited hyperacute practicality
APT-MRI	Lesion-level pH-weighted mapping	Standardization challenges	5–6	Quantitative robustness
*Ex vivo* nanosensors (e.g., SERS/PRET)	Microscale pH sensitivity	No *in vivo* lesion-wide use	2–3	Poor clinical deployability
Fluorescent pH probes	High sensitivity, rapid response	Limited penetration depth	3–4	BBB delivery and biosafety
pH-responsive MRI probes	Spatially resolved pH-triggered contrast	Low clinical maturity	4	Retention and safety
Multimodal pH-responsive probes	Rich multi-parameter readout	High system complexity	3–4	Standardization and regulation

## 5 Translational challenges, perspectives, and roadmap

### 5.1 The translational pathway: challenges and strategies

**FIGURE 4:**
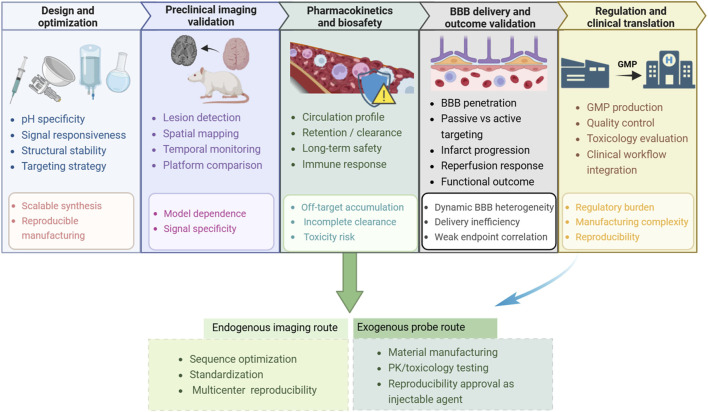
The translational pathway for pH-sensing technologies. The diagram outlines the major stages from probe or platform design to clinical implementation and highlights representative barriers at each step, including scalable synthesis, biosafety and pharmacokinetic constraints, blood–brain barrier delivery efficiency, outcome-oriented validation, and regulatory burden. Distinct translational routes for endogenous imaging approaches and exogenous probe-based systems are also indicated.

Although recent advances in pH-sensitive imaging have substantially expanded the ability to detect ischemia-associated acidosis, the path from experimental validation to clinical implementation remains complex. The translational challenge is not simply whether a technique can detect pH alterations, but whether it can do so with sufficient safety, reliability, reproducibility, and clinical relevance to influence stroke management. In this context, the major barriers extend beyond imaging performance itself and include pharmacokinetics, biosafety, delivery across the blood–brain barrier, outcome validation, and regulatory feasibility. As summarized in Figure 4, this translational pathway extends from probe or platform design to clinical implementation, with distinct bottlenecks emerging at different stages of development and validation. These barriers are especially important in ischemic stroke, where limited therapeutic windows, heterogeneous perfusion, and the need for reliable lesion-level interpretation impose particularly strict requirements on both biosafety and detection efficiency.

A central translational issue, particularly for probe-based systems, is their pharmacokinetic and safety profile. Many pH-responsive nanoprobes are designed to accumulate in ischemic tissue and remain stable until exposed to acidic microenvironments, but this also raises concerns regarding off-target distribution, prolonged tissue retention, and uncertain long-term fate. Inorganic components such as CdSe, PbS, Gd-based agents, and iron oxide nanoparticles may offer strong imaging performance, yet they also introduce risks related to heavy-metal toxicity, long-term deposition, or incomplete clearance (Yang et al., 2017; Yang et al., 2016; Cheng et al., 2021). Even when biodegradable carriers are used, their degradation rate, metabolic by-products, and elimination routes must be carefully characterized. Accordingly, translational progress depends not only on signal responsiveness but also on whether the probe exhibits a predictable circulation profile, acceptable biosafety, and controllable clearance behavior.

Beyond safety and retention, effective delivery to ischemic brain tissue represents a second major obstacle. In principle, blood-brain barrier disruption during stroke may facilitate probe entry, but this process is temporally heterogeneous and regionally variable. Passive accumulation therefore, cannot be assumed to be reliable across different ischemic stages or lesion types. Active targeting strategies may improve tissue selectivity, yet they also introduce additional complexity in probe design and may remain sensitive to the dynamic pathophysiology of the hyperacute period. Thus, the challenge is not merely how to increase probe delivery, but how to ensure that delivery occurs within a clinically meaningful time window and with sufficient specificity to support lesion characterization.

A further limitation in the current literature is the insufficient linkage between pH-related signals and robust biological or clinical outcomes. Detecting tissue acidosis is not, by itself, sufficient to justify translational relevance. For pH-sensitive imaging to contribute meaningfully to stroke grading, derived parameters must be shown to correlate with infarct progression, final infarct volume, tissue salvage after reperfusion, hemorrhagic transformation risk, and long-term neurological recovery. This requirement is especially important for distinguishing between visually impressive probe activation and truly informative metabolic biomarkers. Future studies should therefore prioritize outcome-oriented validation in both small- and large-animal models, with particular attention to reproducibility and longitudinal follow-up.

Finally, the translational pathways of endogenous imaging methods and exogenous probe-based systems differ substantially. Endogenous approaches such as APT-MRI or MRS are more readily aligned with existing imaging infrastructure and may therefore reach clinical testing through technical optimization, sequence standardization, and multicenter reproducibility studies. By contrast, injectable pH-responsive probes face a more demanding route that includes material manufacturing, quality control, pharmacokinetic testing, toxicology evaluation, and regulatory review similar to that required for new contrast agents or theranostic products. This divergence helps explain why some probe-based technologies may show compelling experimental performance while still remaining far from routine clinical adoption.

Taken together, the translational pathway of pH-sensitive stroke imaging depends on more than technical innovation alone. Progress will require coordinated advances in probe engineering, safety profiling, timed and targeted delivery, outcome-based validation, and regulatory strategy. Only by addressing these interconnected challenges can pH-sensitive approaches move from proof-of-concept imaging toward clinically meaningful applications in stroke stratification and management.

### 5.2 Perspectives and roadmap

In view of these challenges, future progress in pH-sensitive stroke imaging should be guided not only by improvements in signal performance, but also by the development of strategies that are quantitatively interpretable, biologically validated, and clinically deployable. At present, endogenous MRI-based approaches, particularly APT-MRI, appear to represent the most realistic near-term pathway toward clinical translation, because they can be integrated more readily into existing imaging workflows and avoid the additional regulatory burden associated with injectable probe systems. (Zheng and Wang, 2021; Geng et al., 2010; Santos-Díaz and Noseworthy, 2020). However, endogenous methods alone may not fully satisfy the need for highly activatable, strongly amplified, or multimodal pH-sensitive readouts. For this reason, probe-based technologies remain important as a longer-term innovation pathway, especially where higher sensitivity, stronger functional contrast, or integrated multi-parameter sensing may be required.

One important priority for future research will be the establishment of quantitative and clinically interpretable pH-related metrics. Rather than treating tissue acidosis as a simple binary signal, future studies should determine which parameters—such as minimum lesion pH, acidosis volume, core–penumbra pH gradients, or pH recovery after reperfusion—show the strongest association with tissue fate and clinical outcome (Harston et al., 2015; Heo et al., 2017; Tietze et al., 2014; Heo et al., 2023). Only when pH-related measurements can be translated into reproducible and outcome-relevant variables will pH-sensitive imaging move from descriptive visualization toward meaningful stroke grading.

A second priority is the integration of pH information with other dimensions of ischemic tissue assessment. In practice, the metabolic information reflected by pH is unlikely to be most useful in isolation. Its greatest value may emerge when combined with diffusion and perfusion imaging, oxygenation-related signals, metabolic biomarkers such as lactate, and clinical severity scores. Such integration may allow a more comprehensive characterization of ischemic tissue status, including the degree of metabolic failure, the likelihood of tissue salvage, and the probable response to reperfusion therapy.

**FIGURE 5:**
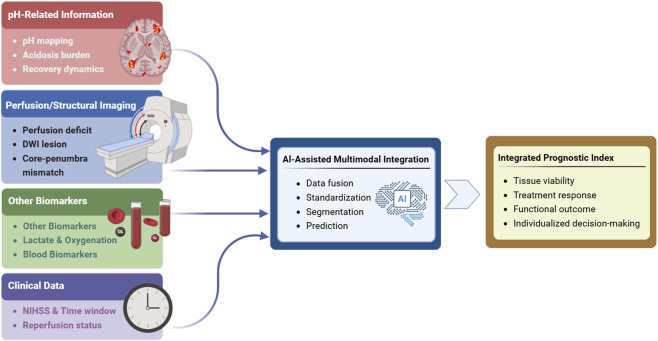
Towards an integrated multi-parameter stroke assessment framework. The schematic shows how pH-related data may be integrated with imaging, biomarker, and clinical information through AI-assisted multimodal analysis to generate a comprehensive prognostic index for ischemic stroke.

Artificial intelligence is likely to play an important role in this process. As pH-sensitive imaging becomes more complex and multimodal, AI-based methods may assist with noise reduction, quantitative standardization, lesion segmentation, and the fusion of heterogeneous datasets across imaging and clinical domains. In this context, a longer-term goal may be the development of a dynamic metabolic–physiological stroke index, in which pH-related information is incorporated together with structural, perfusion, and clinical variables to support individualized decision-making in the hyperacute stage. As illustrated in Figure 5, the future clinical value of pH-sensitive imaging will likely depend on its integration with perfusion imaging, complementary biomarkers, and clinical variables through AI-assisted multimodal analysis, ultimately enabling a more comprehensive prognostic framework for ischemic stroke.

Overall, the future of pH-sensitive stroke imaging will likely depend on balancing innovation with translational realism. Endogenous MRI-based strategies currently provide the clearest short-term route toward clinical implementation, whereas probe-based systems remain important for expanding sensitivity, specificity, and multimodal functionality. The ultimate objective is not simply to visualize acidic tissue, but to convert acidosis into a measurable, interpretable, and clinically actionable indicator of ischemic severity, tissue viability, and therapeutic responsiveness.
