# Correction: pH-sensitive probes for ischemic stroke: advancing early detection and accurate grading

**DOI:** 10.3389/fmolb.2026.1906875

**Published:** 2026-07-09

**Authors:** Chen Zou, Lijun Wang, Yue Wu, Pengfei Yang, Xianfu Meng, Jianmin Liu, Hongjian Zhang

**Affiliations:** 1 School of Health Science and Engineering, University of Shanghai for Science and Technology, Shanghai, China; 2 Oriental Pan-Vascular Devices Innovation College, University of Shanghai for Science and Technology, Shanghai, China; 3 Neurovascular Center, Changhai Hospital, Naval Medical University, Shanghai, China; 4 Department of Nuclear Medicine, Changhai Hospital, Shanghai Key Laboratory of Nautical Medicine and Translation of Drugs and Medical Devices, Navy Medical University, Shanghai, China; 5 Department of Materials Science and State Key Laboratory of Molecular Engineering of Polymers, Academy for Engineering and Technology, Fudan University, Shanghai, China

**Keywords:** early detection, ischemic stroke, metabolic imaging, pH-based grading, pH-responsive nanoprobes, tissue acidosis

Incorrect funding

Missing

The funder Shenzhen Research Foundation, KJZD20240903103304006 to Hongjian Zhang was erroneously omitted.

The original article has been updated.

